# Modality-specific attractor dynamics in dyadic entrainment

**DOI:** 10.1038/s41598-021-96054-8

**Published:** 2021-09-15

**Authors:** Mattia Rosso, Pieter J. Maes, Marc Leman

**Affiliations:** Department of Art, Music and Theatre Sciences, IPEM Institute for Psychoacoustics and Electronic Music, Ghent University, Ghent, Belgium

**Keywords:** Social behaviour, Social neuroscience, Cooperation, Motor control, Sensorimotor processing

## Abstract

Rhythmic joint coordination is ubiquitous in daily-life human activities. In order to coordinate their actions towards shared goals, individuals need to co-regulate their timing and move together at the collective level of behavior. Remarkably, basic forms of coordinated behavior tend to emerge spontaneously as long as two individuals are exposed to each other’s rhythmic movements. The present study investigated the dynamics of spontaneous dyadic entrainment, and more specifically how they depend on the sensory modalities mediating informational coupling. By means of a novel interactive paradigm, we showed that dyadic entrainment systematically takes place during a minimalistic rhythmic task despite explicit instructions to ignore the partner. Crucially, the interaction was organized by clear dynamics in a modality-dependent fashion. Our results showed highly consistent coordination patterns in visually-mediated entrainment, whereas we observed more chaotic and more variable profiles in the auditorily-mediated counterpart. The proposed experimental paradigm yields empirical evidence for the overwhelming tendency of dyads to behave as coupled rhythmic units. In the context of our experimental design, it showed that coordination dynamics differ according to availability and nature of perceptual information. Interventions aimed at rehabilitating, teaching or training sensorimotor functions can be ultimately informed and optimized by such fundamental knowledge.

## Introduction

Humans are in remarkable ways influenced by what they hear and see, especially when they perceive other people moving. The exposure to rhythmic behaviors attracts individuals to fall in sync with one another, and the phenomenon can be observed at the level of dyads, small-groups and large crowds^1^. For instance, when two people walk or dance together, they show a natural tendency to synchronize their steps^2–4^. When an audience applauds at the end of the concert, the auditory scene goes through intermittent periods of synchronized clapping^5^. When marching bands cross each other on the street, it requires conscious effort for them to avoid getting rhythmically entrained^6^.

Rhythmic joint coordination is ubiquitous in daily-life human activities, from the simplest form of unintentional synchronization^7^ to complex tasks such as musical performance^8^. The way different rhythms organize into orderly coordinated patterns over time implies co-regulated actions in response to rhythms produced by other agents. Characteristic for this co-regulation is that action patterns are often spontaneously attracted towards stable synchronization, via a process called *entrainment*^9^. ‘Coordination dynamics’ has been proposed as a theoretical framework to understand the generic organizational principles that underlie the coordination of coupled rhythmic units^7,9–12^. Central to the theory is that the coordination of multiple units over time is the result of a dynamic balance between keeping the own rhythm (competition) and moving together on the collective level (cooperation)^13^. Such idea resonates in earlier work by von Holst^14^, who termed the ‘maintenance effect’ versus the ‘magnet effect’ in his study of coordination in animal behavior. When intrinsic and external rhythms are incongruent, rhythmic units engage in a dynamic competition-cooperation process.

When it comes to rhythmic interactions within a dyad, this dynamic balance can be influenced by manifold variables, such as preferred tempo^15^, empathy^16^ and social factors^17^. However, among all possible sources of variability, informational coupling between individuals stands out as the most fundamental condition for interpersonal coordination. The essential minimal requirement for human interactions to occur is that individuals mutually exchange information through one or more sensory channels. Although experimental interactive scenarios necessarily imply a determined sensory modality^18–20^ and possibly cross-modal interactions^4^, the role of competing sensory modalities remains overlooked in the literature. To this date, whether and how coordination dynamics depend on the nature of available perceptual information requires further systematic investigation.

The aim of the present study is to develop a dynamic experimental paradigm, and apply it for studying modality dependencies of competition-cooperation dynamics in dyadic entrainment. The paradigm involves a unimanual tapping task, where two individuals are exposed to minimally different tempi and reciprocally coupled in visual and auditory modalities across experimental conditions. The procedure implies a tension between competition and cooperation processes in the dyadic interaction, and is meant to return an empirical layout of its dynamics across sensory modalities.

The core idea underlying the proposed experimental paradigm is to induce a competition between the intrinsic rhythm of an individual’s actions and the rhythm produced by another person. During a unimanual tapping task, two partners are exposed to two metronomes in different modalities (visual and auditory). The metronomes assigned to the partners slightly differ in frequency, resulting in a gradual increasing (0 to π rad) and decreasing (π to 0 rad) of absolute relative phase over multiple cycles. Accordingly, for each person, a corresponding cyclical de-phasing of the assigned metronome and partner’s metronome unfolds over time. With this simple expedient, we implemented a completely predictable system of two oscillators which deterministically revisits the same states throughout 10 consecutive cycles. An audio file showing metronomes’ behavior in available in the *Supplementary materials.*

**Figure 1:**
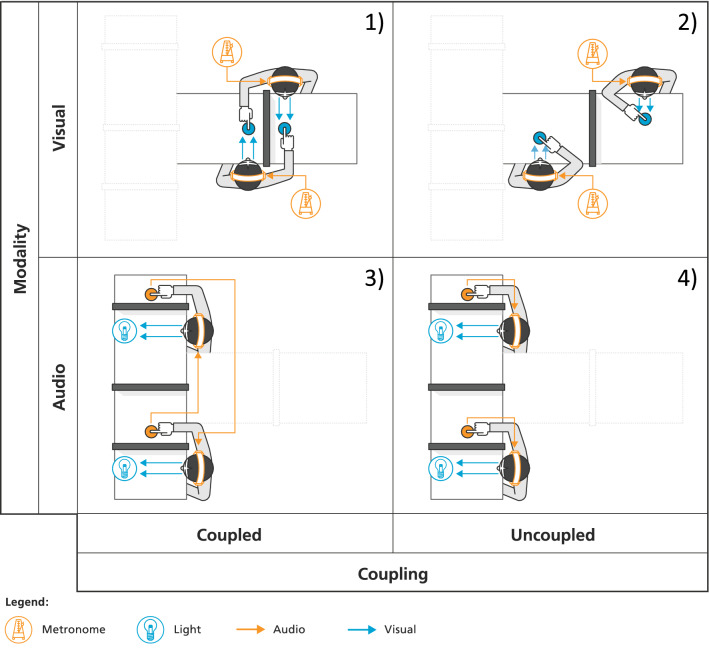
Experimental design. The study was designed in a Modality (Visual, Auditory) x Coupling (Coupled, Uncoupled) factorial structure, resulting in the following conditions. 1. *Visually Coupled*. Participants tapped along with an auditory metronome, while looking at the partner’s hand tapping. The view of their own hand was hidden by a screen placed on the table. They were explicitly asked to neglect the partner’s movements and focus on following their own metronome. 2. *Visually Uncoupled* (*control*). Participants tapped along with an auditory metronome, while looking at their own hand. The view of the partner’s hand is hidden by a screen placed on the table. 3. *Auditorily Coupled*. Participants tapped along with a flickering LED, while hearing the sonification of the partner’s tapping. The view of all the hands was hidden by screens placed on the table. They were informed that the sounds they will hear are produced by the partner, and were explicitly asked to neglect them and focus on following their own metronome. 4. *Auditorily Uncoupled* (*control*). Participants tapped along with a flickering LED, while hearing the sonification of their own tapping. The view of all the hands was hidden with screens placed on the table.

When the partners are informationally coupled, they are exposed to two competing rhythms. The first one is the *intended* rhythm: participants are explicitly instructed to synchronize to their assigned metronome. The second rhythm is the *coupling* rhythm, which is produced by the partner and perceived in another sensory modality. Thus, when the members of the dyad follow their own auditory metronome, they are exposed to visual information from the partner (visual coupling). Alternatively, when the members of the dyad follow their own visual metronome, they are exposed to auditory information from the partner (auditory coupling). The same task performed in absence of informational coupling provides the control conditions for each modality, and a baseline to assess the impact of coupling on spontaneous dyadic entrainment. In presence of informational coupling, a cross-modal incongruency takes place between intended and coupling rhythms. The experimental design is schematized and described in Fig. 1.

In the framework of coordination dynamics, the concept of *relative phase* (ϕ) functions as a collective variable to describe co-regulation of timing. When competition dominates the interaction, rhythmic units may fully keep their own intrinsic frequency, whereas they may be fully attracted towards each other as cooperation prevails. The former case would typically result in an evenly spread distribution across ϕ (0 to 2π radians). In the latter case, the distribution would exhibit peaks over the so-called *attractor* regions, where rhythmic units are locked in temporally stable state. In human behavior, the intrinsic dynamics of the collective variable ϕ are well characterized by a layout of attractive and repelling fixed points, wherein in-phase (0 radians) and anti-phase (π radians) are the dominant stable modes of coordination. The Haken-Kelso-Bunz (HKB) model mathematically describes how phase transitions from one spatiotemporal pattern to another take place when the system is pushed beyond its equilibrium state^21^. The model points at the in-phase mode as the point where the system ultimately tends to reach stability.

**Figure 2:**
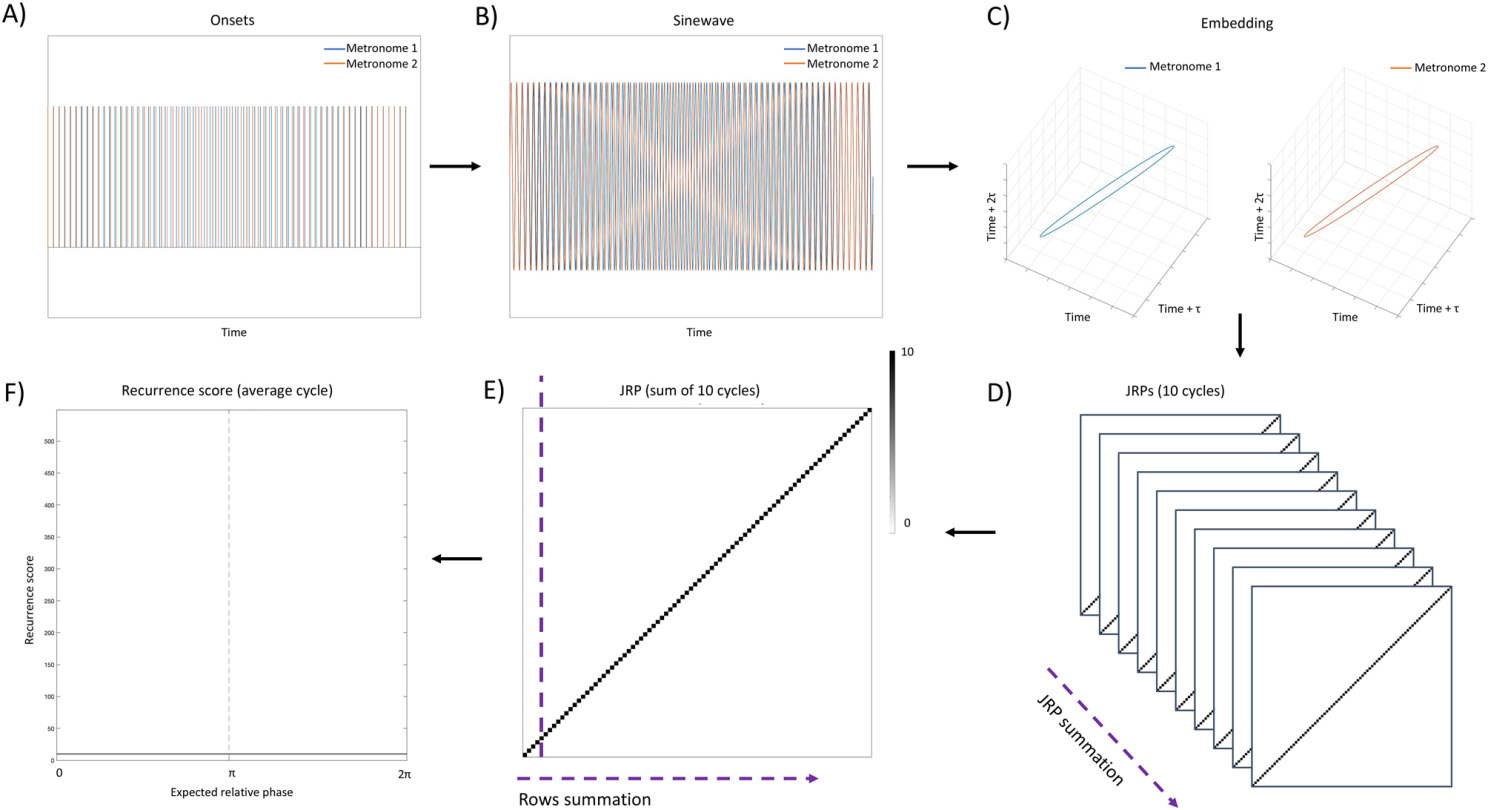
Analysis pipeline. The procedure is here presented as applied to the timeseries generated by the metronomes’ onsets, showing dyadic behavior under the ideal condition of perfect compliance with the task. (**A**) Starting from discrete onsets, (**B**) we interpolated each timeseries with a sine function to model the behavior as a system of coupled oscillators^54^ and (**C**) separately embed them in their respective phase-spaces for JRPs computation^22^. From the representation of one full cycle of the metronomes’ behavior, it is evident how the two oscillators smoothly de-phase and get back in-phase at the end of the cycle. (**D**) JRPs were computed of each individual cycle and (**E**) summed up, returning a 2-D matrix of the density of recurrence points over the trial structure. Finally, by looping over the column and summing all the rows of the matrix, (**F**) we compressed the representation into a 1-D timeseries of the total count of recurrences over the cycle structure. The presence of diagonal lines dominating the JRPs is exactly the configuration expected by a deterministic system^22^, and when compressed in the timeseries format result in a flat horizontal line with small periodic fluctuations (due to the down-sampling performed on the sinewaves prior to embedding, which was necessary to make JRPs calculations computationally feasible). Applying the pipeline to human dyads allowed us to map the local variations of their coupling strength over the expected relative phase, resulting in the picture of the ‘attractor landscape’^34^.

**Figure 3:**
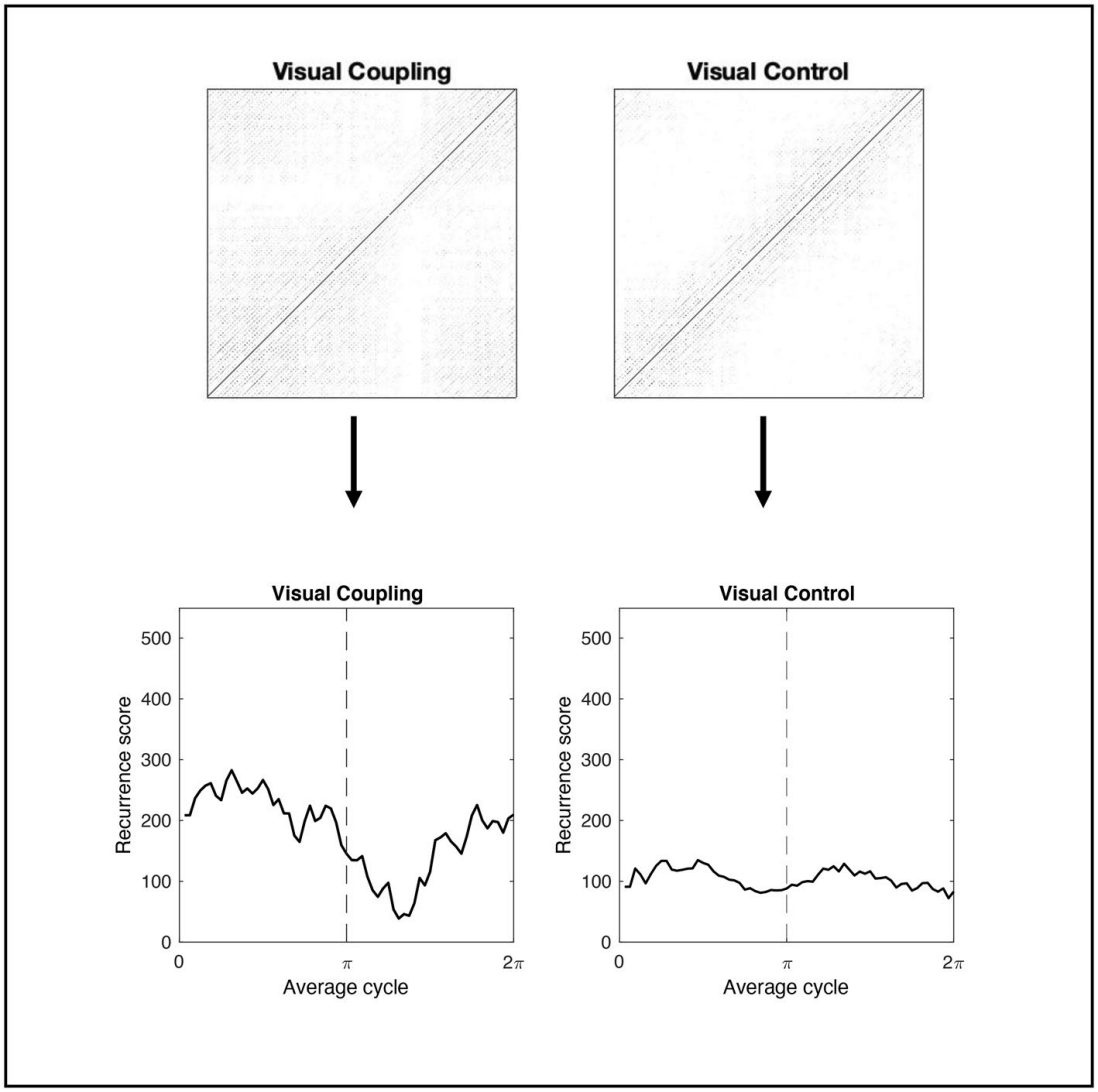
Recurrence score over average cycle. The sum of 10 JRPs from Visual Coupling and Visual Control conditions are compressed to a 1-D recurrence score timeseries. It is evident how visual coupling modulates the density of recurrence point and its distribution over the average cycle in this representative dyad (#5). Compared to the control condition, the recurrence score was higher on average and exhibited local maxima at the extremities of the cycle (i.e., in proximity of the in-phase attractor point).

Crucially, the behavior of the ‘drifting metronomes’ recursively guided the dyads through the whole range of ϕ values—and over its respective attractor regions. Based on a joint recurrence analysis of the system’s dynamics^22^, we computed a recurrence score as a metric of dyadic coupling strength and tracked its evolution over time (for a visual representation of the processing pipeline, see Figs. 2 and 3). By these means, we obtained an empirical attractor layout descriptive of the competition-cooperation dynamics within the dyads. The cooperation process (i.e., entrainment to the partner’s coupling rhythm) was expected to dominate the conflict around in-phase (0 radians) and anti-phase (π radians) points, leading to significantly higher recurrence score in presence of informational coupling and a significant modulation around attractor regions.

Given the time-varying nature of the recurrence score as a response variable, we needed a method to model its local variations over the course of the metronomes’ cycles. We opted then for a statistical model which allowed us to assess the effects of our experimental manipulations over the entire unfolding of the interaction, and to avoid segmentation and multiple comparison while respecting the temporal structure of the data^23,24^. In addition to the inferences made possible by linear models based on intercepts, such as ANOVAs, our solution allowed to assess the effect of categorical predictors on the temporal profile of the response variable. In order to complement our observations at the dyadic level, we used the same framework to analyze the evolution of individual rhythmic behaviors over the course of the task.

## Results

### Growth curve analysis

^25^ was used to analyze the evolution of recurrence score over the course of the expected relative phase between partners (i.e., from 0 to 2π). The curves were calculated as the average of the 10 consecutive metronomes cycles in each experimental condition, and modeled with 2nd order orthogonal polynomials and fixed effects of Coupling and Modality on the polynomial terms. The uncoupled (control) conditions were treated as a baseline for contrasting the levels of the Coupling factor, and the audio conditions were treated as a baseline for the Modality factor.

**Figure 4:**
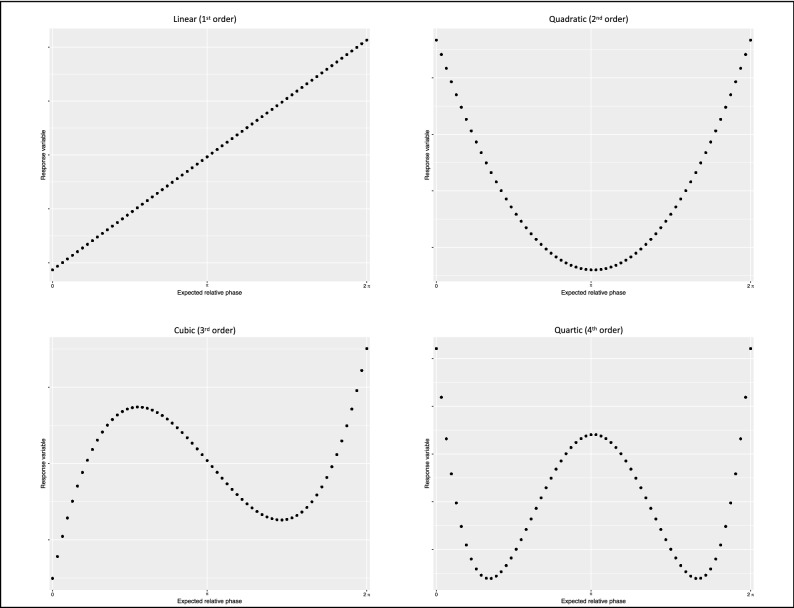
Orthogonal polynomials. The time-varying dimension of a response variable over the metronomes cycle can be modelled with higher-order polynomials. In our model, intercepts correspond to the overall average of the recurrence score: the main effects of categorical predictors indicate global differences across experimental conditions independently from the temporal profile of the response variable. On the other hand, effects of categorical predictors on the polynomial terms indicate the local effect of attractor points on the x-axis. For the linear component (1st order), the parameter estimate corresponds to the slope of the line; for the quadratic component (2nd order), it corresponds to the parabolic curvature; for the cubic and quartic components (3rd and 4th order), it corresponds to the sharpness of the peaks on the inflexion points. The order of the polynomial is ideally chosen based on hypothesis and on the nature of the data, should be confirmed by the empirical data, and should be allow a straightforward interpretation of the effects^25^. In our case, orders from 2 to 4 seem suited candidates for modelling different possible attractor landscapes, given the symmetric measure of the cycle and inflection points around in-phase and anti-phase regions. A full model based on orthogonal polynomials includes all terms up to the chosen order.

In the context of orthogonal polynomials, a flat line can be considered a pure intercept and a ‘zero-order’ polynomial, in the sense that it exhibits zero changes in any direction. If the recurrence score was time-invariant, it would appear as a flat line indicating complete dominance of the competition process between rhythmic units, which would pursue their individual intended rhythms. On the other hand, significant changes of direction would indicate that the interaction is systematically modulated by the temporal structure of the task: around attractor points, the cooperation process would take over and the rhythmic units would be attracted towards the coupling rhythm. Figure 4 shows how orthogonal polynomials are suited for modelling local variations of the response variable around attractor points.

**Figure 5:**
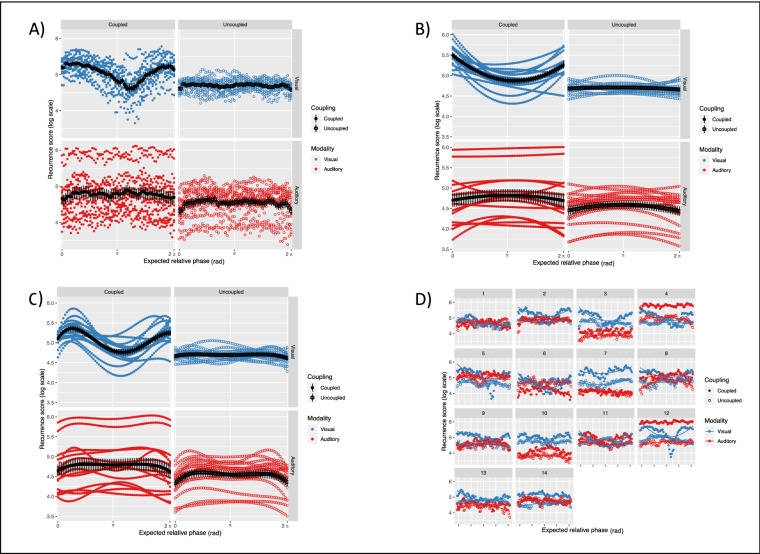
Coupling strength as a function of the attractor landscape. (**A**) Recurrence score (on the log-scale) over the average of 10 cycles, for 14 dyads (colored dots). Grand-average and error bars are represented in black. The reader can visually appreciate the consistency of the “seagull-shaped” pattern in condition of visual coupling and the flat line in the respective control condition. The condition of auditory coupling shows a recurrence score higher on average but no clear pattern over the average cycle, and overall higher variability. (**B**) Recurrence score predicted by our 2nd order polynomial model. The inflection of the parabolic term and the negative slope of the linear term successfully capture the influence of the in-phase attractor points in the visually coupled condition. Arguably, this is the most parsimonious model for our data in the framework of growth curve analysis^25^. (**C**) Recurrence score predicted by our 4th order polynomial model. The quartic fit manages to capture more fine-grained modulations, particularly the local maxima on the inflection points at the extremes of the cycle. The profile recalls the typical ‘seagull-effect’ originally reported in^34^ and discussed in^12^ in the context of bimanual coordination dynamics. However, for the sake of interpretation of parameter estimates, we opted for the simplest possible model: given the absence of a local maximum on the anti-phase point, a single parabolic inflection point was sufficient to explain the effect of visual coupling. (**D**) Within-dyads comparison of the recurrence score over the average trial, across experimental conditions. Dots represent the empirical data, whereas continuous lines represent the full model (up to the 2nd order polynomial) fitted to the data.

Orthogonal polynomials in the model were limited to the 2nd order: based on our hypotheses, we expected quadratic and quartic terms to capture the effects of in-phase and anti-phase attractor points. However, following data inspection and analysis of residuals, we concluded that the quadratic term alone was enough to capture the relevant effect of our experimental manipulation. Figure 5 shows the data with descriptive statistics across experimental conditions, and a comparison between 2nd order and 4th order models. Arguably, the better fit of the quartic term comes with a major cost in terms of model parsimony and interpretation. Our model also included random effects of Dyads on all polynomial terms, and their interactions with the factors: the random effects structure was maximized in order to minimize false alarm rates without substantial loss of power^26^.

As expected, we found a significant main effect of Coupling (*Estimate* = 42.809, *SE* = 18.142, *p* = 0.018) indicating an overall increase of the recurrence score in presence of informational coupling as compared to the uncoupled control conditions, independently from the sensory modality. We also report a significant interaction effect of Coupling and Modality on the orthogonal polynomial terms of Time (Linear: *Estimate* = −84.579, *SE* = 30.867, *p* = 0.006; Quadratic: *Estimate* = 190.320, *SE* = 36.973, *p* < 0.001), meaning that the parameter estimates in coupled conditions significantly differ across visual and auditory modalities. Given the observed major variability in the Auditory level and following inspection of the residuals, we fitted the same model on the natural log-transformed response variable for it provided a considerably better fit, and still found significant main effect of Coupling (*Estimate* = 0.237, *SE* = 0.117, *p* = 0.042) and interaction effects on the polynomial terms of Time (Linear: *Estimate* = −0.534, *SE* = 0.222, *p* = 0.016; Quadratic: *Estimate* = 1.257, *SE* = 0.299, *p* < 0.001). Table 1 shows the fixed effects parameter estimates and their standard errors for log-transformed recurrence score, along with p-values estimated using the normal approximation for the t-values.

**Table 1:** Models’ summary.

Predictors	Recurrence score (N = 14)		
	*Estimates*	*SE*	*p*
Time	− 0.037	0.113	0.739
Time^2^	− 0.360 *	0.150	**0.017**
Modality	0.155	0.117	0.184
Coupling	0.237 *	0.117	**0.042**
Time: Modality	− 0.021	0.157	0.893
Time^2^: Modality	0.269	0.212	0.202
Time: Coupling	0.011	0.157	0.942
Time^2^: Coupling	0.055	0.212	0.793
Modality: Coupling	0.118	0.165	0.475
Time: Modality: Coupling	− 0.534 *	0.222	**0.016**
Time^2^: Modality: Coupling	1.257 ***	0.299	** < 0.001**
	*p < *0.05*	**p < *0.01*	***p < *0.001*

Predictors	Phase error (N = 28)		
	*Estimates*	*SE*	*p*
Time	− 0.177	0.165	0.283
Time^2^	− 0.172	0.190	0.364
Modality	− 0.230 **	0.080	**0.004**
Coupling	0.156	0.080	**0.051**
Time: Modality	0.138	0.218	0.526
Time^2^: Modality	0.027	0.264	0.917
Time: Coupling	0.293	0.218	0.179
Time^2^: Coupling	0.191	0.264	0.467
Modality: Coupling	0.183	0.113	0.106
Time: Modality: Coupling	− 0.404	0.309	0.190
Time^2^: Modality: Coupling	1.058 **	0.373	**0.004**
	*p < *0.05*	**p < *0.01*	***p < *0.001*

All significant effects are marked with an asterisk, and the associated * p*-values are highlighted in bold. We reported a significant main effect of Modality and significant 3-way interactions with linear and quadratic functions of Time. In the framework of orthogonal polynomials, coefficient estimates represent a measure of effect size^25^: the intercept represents the average increase in the response variable contrasted to the baseline (i.e., the Uncoupled level), the slope of the linear term represents the change in the response variable per unit of time, and the quadratic term represents the steepness of the parabolic inflection. For the recurrence score, the natural exponential of the coefficient estimates should be used for interpretation purposes, given that the response variable was transformed to the log-scale. The significant correlation with the quadratic function of time was not discussed in the text, for it does not have relevance in the context of the experimental design.

**Figure 6:**
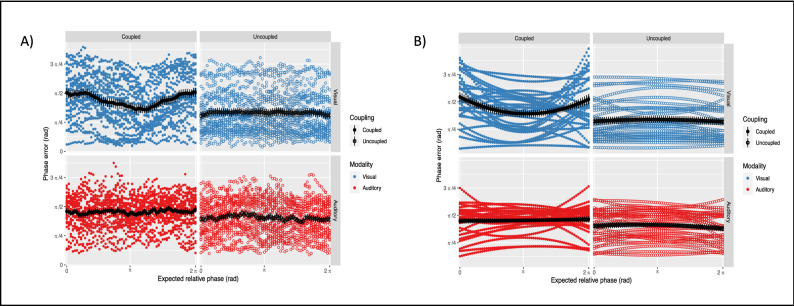
Individual synchronization as a function of the attractor landscape. (**A**) Phase error over the average of 10 cycles, for 28 participants (colored dots). Grand-average and error bars are represented in black. The reader can visually appreciate how individual rhythmic behaviors track the course of the recurrence score, supporting the idea of a dynamic balance between cooperation and competition processes. As the coupling between partners dominates the interaction, the magnitude of the phase error increases accordingly. (**B**) Phase error predicted by the 2nd order polynomial model. The "seagull-shaped" pattern observed in the visually coupled condition could be well modelled by the inflection of the parabolic term alone. Differently from the recurrence score, phase error exhibited a symmetric profile without significant interactions with the linear term.

The same model was then fitted to the phase error of the individual participants. The response variable was computed as the absolute phase difference between the finger-tapping and the metronomes timeseries, to quantify individual synchronization behavior. We found a significant main effect of Modality (*Estimate* = −0.230, *SE* = 0.080, *p* = 0.004), indicating that the assignment to visual metronomes resulted in higher phase error. Furthermore, the quasi-significant main effect of Coupling (*Estimate* = 0.156, *SE* = 0.080, *p* = 0.051) confirmed that informational coupling with the partner negatively affected the synchronization with the assigned metronome. When looking at the evolution of the phase error over time, the interaction effect of Coupling and Modality on the quadratic term of Time (*Estimate* = 1.058, *SE* = 0.373, *p* = 0.004) revealed that the temporal profile across conditions is coherent with the one observed for the recurrence score (see Fig. 6).

In the context of growth curve analysis^25^, the parameter estimates provide a measure of effect size of straightforward interpretation for linear and non-linear changes over time as long as the order is not too high: above the 4th order, results become hardly interpretable and the risk of over-fitting the data increases. With the interaction effect of Coupling and Modality factors on the polynomial terms, we could quantify modality-specific effects of informational coupling on the evolution of the recurrence score and the individual synchronization performances. The results strongly support our predictions of spontaneous dyadic entrainment in presence of informational coupling and, crucially, show that its temporal dynamics are modality-dependent.

## Discussion

The present study investigated the dynamics of dyadic entrainment, and more specifically how they depend on the sensory modality mediating informational coupling. An experimental paradigm was designed to dynamically manipulate the expected relative-phase between participants, while informational coupling was manipulated in visual and auditory modalities across conditions. At the global level, we wanted to prove that when intended and coupling rhythms are at odds, a spontaneous co-regulation of timing occurs within the dyad at the expenses of the individual intended rhythms. At the local level, we wanted to infer the configuration of a dyadic *basin of attraction*, by modelling the dyadic cooperation process around attractor points^12^. Ultimately, it was possible to evidence a crucial modality-dependency of dyadic entrainment.

In the first place, we reported that the mere presence of informational coupling led to spontaneous entrainment in dyads engaged in a rhythmic task. As long as two partners perceived each other, their rhythms were ‘attracted’ towards each other. The effect was quantified as a significant increase of the average recurrence score, compared to the baseline provided by the de-coupled conditions. Remarkably, such manifestation of self-organizing behavior^12,27^ emerged in the context of a task where subjects were explicitly instructed to ignore each other’s actions, pursuing independent uncoupled behaviors cued by their own reference. Even though the observation of spontaneous dyadic entrainment was reported in self-paced^19,28^, cued^29,30^ and joint improvisation tasks^31–33^, our procedure explicitly implemented rhythmic incongruency to investigate the competition-cooperation dynamic balance latent to the system’s dynamics^13^. When competition prevails, rhythmic units manage to keep the assigned (intended) rhythm, whereas when cooperation takes over, rhythmic units undergo a (spontaneous) co-regulation of timing and move together on a collective level of coordinated behavior. Overall, both visual and auditory coupling led to spontaneous dyadic entrainment despite the instruction of synchronizing with the assigned metronome. Moreover, the shift towards a cooperation process occurred at the expenses of the competing individual behaviors, as indicated by the significant increase of the average phase error in coupled conditions.

Beyond a global measure of coupling, the continuous de-phasing implemented in the ‘drifting metronomes’ paradigm allowed us to quantify local variations of recurrence score as a function of the expected relative phase between partners. The balance between cooperation and competition processes is expected to be dynamic, in the sense that it may vary over time and is constrained by the intrinsic dynamics of the system. Dyadic entrainment was expected to occur more strongly and more consistently around attractor regions, located around the in-phase and anti-phase points of the metronomes’ cycles^21^. With our approach, we achieved to return an empirical ‘attractor landscape’^34^ and to contrast it across conditions of visual and auditory coupling. Our findings strongly point towards a modality-dependency of dyadic entrainment (see Fig. 5), which in turn is reflected in the individual synchronization with the metronomes (see Fig. 6). The interaction effect of Coupling and Modality on the linear and quadratic terms of Time statistically confirms that the temporal modulation of the recurrence score is an exclusive property of visually-mediated coupling, with a remarkable consistency across dyads. In such condition, the recurrence score exhibits maximum values around the in-phase point at the start of the metronomes cycle, gradually decreases as the dyad leaves the attractor zone, reaches its minimum passed the anti-phase point, and finally increases towards maximum values as it enters the in-phase zone at the end of the cycle. On the other hand, the individual phase error exhibits a more symmetrical parabolic modulation, with only interaction on the quadratic term of Time reaching significance.

Selectively for the recurrence score, we report here an effect of directionality in the visually coupled condition: dyads slowly de-couple as they leave the in-phase point, whereas they couple more rapidly as they approach the same point. The negative slope of the linear term indicates that dyads tend to be more coupled in the first portion of the cycle, namely before passing the anti-phase point. The observation is suggestive of a hysteresis effect, which characterizes transitions from one attractor to another in dynamical systems^12^. In interlimb and interpersonal coordination, non-linear transitions from anti-phase to in-phase coordinative modes are typically observed as the system is pushed towards instability^18,35,36^. This can be experimentally induced in an experimental setting by increasing the frequency of the rhythmic units as control parameter. In our paradigm, we could appreciate that increasing the metronomes’ relative phase away from the in-phase point, the attractor exerted a long-lasting ‘pull-back’ effect. This was followed by a more abrupt ‘push-forward’ when transitioning over the anti-phase point, as the expected relative phase decreased. Even though the metronomes’ frequencies were not manipulated, their continuous de-phasing resulted in repeated transitions from in-phase to anti-phase regions and vice versa, as the dyad explored the attractor landscape.

In condition of auditory coupling, the recurrence score did not exhibit any significant variation over time: the measure was locally independent from the proximity of attractor points. Nevertheless, we still observed a significant global increase of recurrences compared to the baseline. Previous evidence shows that phase shifts in auditory sequences elicit a phase-correction response in participants instructed to tap in synchrony to isochronous sequence of flashing lights^37^. Auditory-driven phase corrections are prone to occur even when shifts are highly irregular^38^ and at different relative phases values, with overall high interindividual variability^39^. Similarly, in the context of our paradigm, sounds generated by the partner exerted attraction on the self-generated taps over the whole course of the metronomes’ cycle, regardless of the expected relative-phase value.

Finally, both control conditions resulted in a flat horizontal line below the average of the respective coupled conditions, as expected. It is worth noting the higher variability in Audio conditions, arguably due to the task of synchronizing to visual metronomes. From lower recurrence score in the control condition, we can conclude that dyads performed globally worse in synchronizing to visual cues as compared to auditory cues. This is in line with previous evidence on modality-dependent synchronization skills^40,41^, and backed by our concomitant observation of higher phase errors when visual metronomes were presented. Taken together, our findings support our hypothesis of modality-dependent dynamics, suggesting that auditorily-mediated entrainment is considerably less sensitive to the basin of attraction when compared to its visual counterpart.

Although interpersonal coordination can be described at the collective level as a self-organizing process^12,27^, humans ultimately entrain to each other via mutual adaptation of individual action-perception loops^19,20^. Therefore, an exhaustive account for dyadic entrainment should be capable of bridging the two levels, considering how coordination dynamics depend on the processing of information available in the interaction. Such link is foundational for translating dynamical control principles from intra- to inter-personal levels^18^. Recently, predictive coding^42–44^ was put forward as a plausible theoretical account for coordination dynamics in dyadic behavior^45^. The theory states that the brain is constantly engaged in an optimization process based on Bayesian inference: information sampled from the environment is compared to prior evidence, and the optimization consists of minimizing prediction errors. In the context of interpersonal coordination, such a putative mechanism could underpin the overt manifestations of dyadic entrainment by engaging the action systems of the rhythmic units involved. Let us consider the prediction error as the phase difference between executed and observed movements: as both units minimize the mismatch between the representations of observed and own motor behavior, the dyadic system tends towards a collective minimization of free energy in a stable attractor state^45^. Such interpretation of the dyadic attractor is supported by our results in the context of visually-mediated coupling: as the relative phase between partners increases, the cooperation process between rhythmic units loses strength in favor of competition. In other terms, the tendency of the partners to lock in a stable coordinative state depends on the energy required to correct for the prediction error. As the error increases, the correction becomes more effortful and it becomes easier for the units to pursue independent behaviors.

Crucially, such explanation does not hold for auditorily-mediated coupling. As distinct sensory systems have their unique interface with motor and timing systems^41,46,47^, it is necessary to interpret the results in light of individual information processing. There is arguably a crucial difference in the nature of the perceptual information available to the partners across different modalities. When the coupling is visually mediated, a stream of kinematic information is continuously sampled from the partner's actions^48^, such that the predictive models can be constantly updated at every stage of movement execution. On the other hand, in case of auditory coupling the prediction of the partner's tap is solely based on temporal information about the previous taps, since partners do not have online access to the reciprocal kinematic information. In such condition, the cooperation process still dominates the interaction but no consistent pattern emerges in terms of dynamics as quantified by our method. We propose that the availability of kinematic information through informational coupling is a crucial discriminant that might explain the observed modality-dependence of dyadic entrainment. The idea that prior evidence is built differently across modalities depending on availability of sensory information is consistent with a predictive account for dyadic entrainment.

A final question arises spontaneous: how would the dyad behave if kinematic information was embedded in a sound envelope, and conveyed to the partners via the auditory channel? One limitation of the current work is that we cannot conclude whether dyadic entrainment is modality-dependent in the strict sense or rather dependent on availability of kinematic information. Given the central role attributed to kinematics in predictive accounts for action perception^48^, it is of particular interest to investigate whether continuous sonification of movement parameters^49^ would support the same dynamics observed in presence of visual coupling. Previous work shows that presenting visual stimuli in a way that indicates movement over time, e.g., apparent hand motion^50,51^ or a bouncing ball^40,46,52^, behavioral outcomes of predictive entrainment are improved by the continuous stream of information. We hypothesize that a movement sonification strategy based on this principle could translate the kinematic advantage into auditorily-mediated dyadic interactions, resembling the dynamics here described in condition of visual coupling.

The findings and the questions raised from the present study might have relevant implications for optimizing interventions aimed at rehabilitating, teaching or training sensorimotor functions. Fundamental knowledge about dyadic interactions is what will ultimately inform such optimization. The evidence from the present study points at the advantages of the physical presence of a teacher (or a therapist, or a trainer) for guiding rhythmic movements, possibly integrating kinematic information via sonification strategies^49^.

In conclusion, we want to highlight that the ‘drifting metronomes’ procedure is meant as a methodological contribution for the investigation of interpersonal coordination, in the hope that the scientific community can build upon it with new research questions and experimental designs. We point at its adoption with simultaneous dual-electroencephalography recordings^53^, computational simulations^54^ and replication on pathological populations^55–57^ as potential sources of insight into the fundamentals of dyadic interactions.

## Methods

### Participants

Twenty-eight right handed participants took part in the study (18 females, 10 males; mean age = 29.07 years, standard deviation = 5.73 years). They were randomly paired in fourteen (N = 14) gender-matched dyads, in order to control for any gender bias in the interaction. None of them had history of neurological, major medical or psychiatric disorders. All of them declared not to be professional musicians upon recruitment, although some of them had musical experience. With the exception of one dyad, all participants declared not to know the assigned partner from before the experiment. The experiment was approved by the Ethics Committee of Ghent University (Faculty of Arts and Philosophy) and informed written consent was obtained from each participant. A 15€ coupon was given to all participants as economic compensation for their time.

### Experimental apparatus and procedure

The two partners were sitting across the same table, facing each other. Chairs were provided with an armrest in order to exclude any tactile or proprioceptive coupling due to the table’s vibrations. Each partner was assigned to one pad and instructed to tap on it with the right index finger synchronizing with a metronome. As represented and described in Fig. 1, the metronome was either an auditory cue or a flickering light, depending on the experimental condition. Each partner was cued with a different tempo (1.67 Hz and 1.64 Hz). Aligning the start of the two tracks, the relative phase between the metronomes started at 0º and steadily increased in regular steps of 5.6º. A full cycle took 39 s to be completed. 10 consecutive cycles were performed in each experimental condition. In conditions of informational coupling, participants were instructed to ignore the partner and tap along with the assigned metronome.

A M-Audio M-Track 8 soundcard was used to route independent audio channels to each participant via in-ear plugs. Ableton Live 10 was adopted as main interface for stimuli presentation, to sonify the participants’ finger-taps and to route them in real-time. The same MIDI tracks were used to control the metronomes across conditions, by either triggering an audio sample or a flickering LED. Volume was adapted to every participant before the start of the experiment, and pink noise was regulated up to the point of suppressing any sound other than the auditory stimulation presented via Ableton. Participants were monitored on-line by means of a USB-camera, to make sure they complied with the instructions. No dyad was excluded from the analyses.

A Teensy 3.2 microcontroller was used as serial/MIDI hub in the setup. It was used to detect tapping onsets with 1 ms resolution, based on the analog input from strain gauge pressure sensors installed inside the pads. Every time a metronome onset was presented to a participant, a MIDI message was sent to the Teensy device to log the metronomes timeseries and to control the voltage of the LEDs when needed.

Simultaneous EEG recordings were performed from both partners of the dyads during the whole experiment, but such data were not presented in the present paper. Additional data were collected prior and during the experiment. Prior to the experiment, demographical data were collected; the *Edinburgh inventory* ^58^ was administered to assess the right handedness of the participants; the *Interpersonal Reactivity Index* (IRI) was administered as self-report of empathy and its subscales ^59^; the preferred spontaneous tempo was calculated via 30 s of self-paced finger-tapping on a dedicated smartphone app (www.beatsperminuteonline.com). During the breaks between experimental conditions, all participants provided subjective self-reports on different aspects of the task by expressing agreement on a scale from 1 (“Completely disagree”) to 7 (“Completely agree”) with a custom-made battery of 11 Likert items. Data collected from the questionnaires were not presented in the present paper.

## Data analysis

### Pre-processing

Our raw data consisted of timestamps logged from the Teensy controller with 1 ms resolution for 10 consecutive metronome cycles (390 s in total), and an associated ID for each partner and each metronome. As only form of cleaning, we removed onsets occurring < 350 ms from the previous one: false positives could occasionally be recorded when a participant pushed the pad for too long or accidentally laid the hand on it. The cleaned timeseries were then interpolated with a sine function at 1 kHz sampling rate, providing an estimate of the oscillators’ positions on its cycle with a temporal resolution of 1 ms. Conceptually, the choice of sinusoidal interpolation was supported by recent work on modelling of systems of coupled oscillators in joint finger-tapping studies^54^. Operationally, it guaranteed that all timeseries match in size without any loss of data, which was a requirement for the next steps of our analysis. Timeseries were finally downsampled by a factor of 40 to make the computation of RPs computationally feasible. Different orders of downsampling were tested to make sure that the results do not depend on this choice.

### Phase-space reconstruction

The optimal parameters for the time-delayed embedding were computed for each participant, for the time course of each single metronomes cycle in all experimental conditions. The resulting mean value across all participants was applied to all individual instances. The reason for this approach is that in order to compare the rate of recurrences across conditions at the group level, the embedding procedure must be consistent across participants (e.g., see^60^, for an example of parameter selections in a factorial design). We first selected the delay *tau* of the timeseries (*τ*) as the first local minimum mutual information index^61^ as a function of delay. This approach minimized the timeseries self-similarity, extracting nearly orthogonal components and preventing the attractor from folding over itself^62^. The mean value of *τ* resulted to be 7. Next, we determined the number of embedding dimensions *m* with the method of false nearest neighbor^63^: we progressively unfolded the time series into higher dimensions until the data points did not overlap spuriously, finding a mean *m* = 3. Finally, in order to determine a maximum threshold for counting two neighboring points as recurrent, we selected a *radius* of 10% of the maximal phase-space diameter^22^.

### Joint recurrence plots (JRPs)

Individual recurrence plots where computed as follows:

$${R}_{i,j}\left(\varepsilon \right)=\Theta (\varepsilon - \Vert {x}_{i}- {x}_{j}\Vert )$$

where ε is the neighborhood threshold, ‖ ⋅ ‖ is the Euclidean norm, and Θ is the Heaviside step function. A square matrix was returned from each shadow-manifold in the phase-space, containing 1s for all the instances where the distance ‖ · ‖ was smaller than the threshold ε, and 0s for the remaining elements. The distance was computed with the method of maximum norm. A joint recurrence plot (JRP) was computed for each dyad by overlapping the individual JRPs of the partners pair-wise, and keeping as 1 only the instances where both plots contained a recurrence. The computation of the JRPs was carried out with the *crp toolbox* for Matlab^22^.

The 10 trials (i.e., the metronome cycles) of each experimental condition were aggregated by summing the respective JRPs such that every cell of the 2-D matrix contained the count of times that a recurrence occurred in the same point of the cycle. Finally, we looped over the columns of the matrix summing all the counts contained in the rows, obtaining a 1-D vector recurrence scores which represented a density measure of the instances of coupled behavior over the course of the cycle. The scale of the recurrence score depends on the size of the JRPs and in turn on the embedding procedure, which made it necessary to set the same parameters for the whole sample. In order to stabilize our response variable and avoid over-sampling in view of our statistical model, the resulting timeseries were divided in 64 segments averaging the recurrence score. For the division, we chose the intervals determined by the steps of the slower metronome, as they provided an intrinsic regular subdivision of the experimental runs. All the steps presented so far were carried out in Matlab (see Fig. 2, for a schematic representation). Our approach was preferred over the “windowed” version for JRQA, for the latter would low-pass filter our timeseries and make it impossible to interpret the results. The resulting phase-shift would be dependent on the choice of the window size, hence not reliable for detecting attractor points over the landscape.

### Individual rhythmic behavior

For every participant, phase angle timeseries were computed by linearly interpolating the finger-tapping onsets as a rampwave at 1 kHz sampling rate, and scaling it by 2π. The same procedure was repeated for the metronomes’ onsets. Phase error was calculated as the absolute difference between the participants’ and the assigned metronomes’ timeseries, wrapped to π and averaged within 64 bins like the recurrence score timeseries. This measure was used as response variable to assess the individual rhythmic behavior of the participants, complementing our findings at the level of collective behavior.

### Statistical models

The recurrence score was used as response variable in a mixed-effects model with Modality and Coupling as factors, and Time as a continuous predictor expressed with the indexes of the metronome’s steps (from 1 to 64). Given the non-linear time course observed in the 'Visual Coupling' condition, we adopted the method of orthogonal polynomials^25^ including linear and quadratic functions of Time into our model (see Fig. 5). Dyads and interactions between Dyads and factors were modelled as random effects^26^ to account for the individual variability in synchronization skills and individual susceptibility to coupling across the experimental manipulations. The formula of the full model (up to the 2nd polynomial order) is the following:

$$Recurrence \sim \left(Time+{Time}^{2}\right)*Modality*Coupling+\left(Time+{Time}^{2} \right| Dyad)+ \left(Time+{Time}^{2} \right| Dyad:Modality:Coupling)$$

The same model was fitted to the phase error of individual participants. Here, Subjects and Dyads were modelled as random effects.

$$Error \sim \left(Time+{Time}^{2}\right)*Modality*Coupling+\left(Time+{Time}^{2} \right| Subject:Dyad)+ \left(Time+{Time}^{2} \right| Subject:Dyad:Modality:Coupling)$$

Statistical analyses were carried out in R (version 4.0.3); model fitting was performed with the *lme4* package^64^. All methods were carried out in accordance with relevant guidelines and regulations, based on the references provided in the respective paragraphs.

## Supplementary Information

Supplementary Information 1.

Supplementary Audio 1.

Supplementary Information 2.

Supplementary Information 3.

Supplementary Information 4.

Supplementary Information 5.

Supplementary Information 6.

Supplementary Information 7.

## Data Availability

All data generated during this study are included in this published article (and its Supplementary materials), together with the scripts used to analyze them.
