# Supplementary material for: Modality-specific attractor dynamics in dyadic entrainment
Source: Sci Rep. 2021 Sep 15;11:18355. doi: 10.1038/s41598-021-96054-8 (PMC8443558; doi:10.1038/s41598-021-96054-8)

**Supplementary material for**

Dyadic entrainment. An overwhelming and modality-dependent phenomenon.

Mattia Rosso, Pieter J. Maes, Marc Leman.

**Email:** mattia.rosso@ugent.be

**This document includes:**

Audio files S1

Datasets S1 to S5

Scripts S1 to S2

Functions S1 (GitHub repository)

Questionnaires S1

All supplementary materials are accessible as separate files or via GitHub repositories, and allow the reviewers to replicate the analyses presented in the main manuscript. Furthermore, the Audio file S1 is meant to provide an immediate intuition of how the “drifting metronomes” paradigm unfolds over time.

NOTE. Dataset S3 is provided in .xlsx format, which can be directly used by Script S2 to replicate our statistical analyses in R.

On the other hand, Datasets S1 and S2 are provided in *.mat* format via GitHub repository. The reason is that due to the nature of the dyadic task and the experimental design, the use of .csv of .xlsx is sub-optimal for handling the data in phase of processing. Given we provide Matlab scripts for processing data, readers and reviewers can replicate our procedure step-by-step and test its validity by making use of the .mat Datasets.

**Audio file S1 (separate file).** The file reproduces the 2 metronomes played together over 10 consecutive cycles. Note that this serves for presentation purposes alone, it is meant for the reader to better understand the collective behavior of the metronomes. Participants were actually presented with only one single metronome, and where never exposed to the partner’s metronome. Visual metronomes exhibited the identical de-phasing pattern as the auditory metronomes hereby presented.

**Dataset S1 (GitHub repository).** This Matlab dataset contains the onsets of the participants’ taps, following the ‘debouncing’ procedure described in the *Methods*. The structure contains one entry for each dyad, and is divided in two fields (‘subject1’, ‘subject2’). Each field contains a cell with 4 entries, one for every experimental condition (1-‘Visually Coupled’, 2-‘Visually Uncoupled’, 3-‘Auditorily Coupled’, 4-‘Auditorily Uncoupled’). The dataset is loaded by the Matlab Script S1, and goes through the processing pipeline as descripted in Figure 2 (see main manuscript).

**Dataset S2 (GitHub repository).** This Matlab dataset contains the onsets of the metronomes. They are used to provide a ‘ground truth’ for the expected behavior of the system, and to guarantee the alignment of the behavioral timeseries.

https://github.com/mattiaIPEM/Datasets-pre-processing-

**Dataset S3 (separate file).** This table constitutes the input for statistical analyses in R (Script S2). Data are organized in a longitudinal structure. The table is returned as output from Script S1, at the end of the data processing pipeline (Script S1, line -270).

**Dataset S4 (separate file).** This table contains the self-reports of participants. Likert-items can be found on the first page of the document Questionnaires S1. These data where not included in the statistical analyses presented in the main manuscript.

**Dataset S5 (separate file).** This table contains demographic and miscellaneous data about the participants. Likert-items can be found on the first page of the document Questionnaires S1. These data where not included in the statistical analyses presented in the main manuscript.

**Script S1 (separate file).** This Matlab script contains the whole processing pipeline as described in the main manuscript. All documentation is provided within the script, block after block. All the functions called by the script are contained in the Functions S1 folder. The current script and the functions should be contained in the same folder, and the directory of such folder has to be included at lines -62 and -122.

**Functions S1 (GitHub repository).** The following link directs to a folder containing all functions and toolboxes called by Script S1. The folder must be downloaded and kept in the same directory as the Script and the Datasets.

https://github.com/mattiaIPEM/Functions-S1

Would there be any issue with the access to the repository, the folder can be provided via email by the corresponding author.

**Script S2 (separate file).** This R script runs the statistical analyses as described in the main manuscript. Line-by-line documentation can be found within the script itself. Plots of data, model fit and model residuals are included.

**Questionnaires S1.** This document contains the questionnaires administered to the participants between experimental conditions.


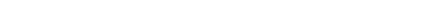

Supplement: Supplementary file 1 — Supplementary Information 1. [file 41598_2021_96054_MOESM1_ESM.docx]
