# Supplementary material for: Modality-specific attractor dynamics in dyadic entrainment
Source: Sci Rep. 2021 Sep 15;11:18355. doi: 10.1038/s41598-021-96054-8 (PMC8443558; doi:10.1038/s41598-021-96054-8)
Supplement: Supplementary file 6 — Supplementary Information 5. [file 41598_2021_96054_MOESM6_ESM.docx]

%%%%%%%%%%%%%%%%%%%%%%%%%%%%%%%%%%%%%%%%%%%%%%%%%%%%%%%%%%%%%%%%%%%%%%%%%%%

% This Matlab script contains the apporach to behavioural data analysis from

% the "drinfting metronomes" paradigm. It consists of the following

% sections:

%

% - Tuning embedding parameters: computation on individual trials for each

% individual in all experimental conditions; the final value consists of

% the Grand-Average to guarantee the same Recurrence Plots size (note

% that paired timeseries must be embedded with the same parameters)

%

% - Preprocessing timeseries: tapping onsets are re-sampled at 1kHz to

% guarantee alignement and 1 ms precision; inter-onset regions are linearly

% interpolated and scaled to 2pi, resulting in a 'sawtooth' phase

% timeseries for calculation of relative phase; the sawtooh timeseries is

% finally converted to a sinusoidal wave for embedding and Joint Recurrence

% Plots (JRPs) computation.

%

% - Computing recurrence score: JRPs are computed for

% each trial taking as input the sinusoidal timeseries; the resulting 10

% JRPs are overlapped and summed; the resulting summed JRP is converted

% to the "recurrence score", i.e. a 1-dimensional timeseries of the

% recurrence count for each position on the (aggregated) trials.

%

% - Output for statistical analyses in R

%

%

%

% Author: Mattia Rosso

% Ghent, 16/10/2020

%%%%%%%%%%%%%%%%%%%%%%%%%%%%%%%%%%%%%%%%%%%%%%%%%%%%%%%%%%%%%%%%%%%%%%%%%%%

%% Settings

% Clear all

clear; close all; clc

% Sampling rate is set to 1000Hz and must not change: data were recorded

% with 1ms time resolution. Change downsampling accordingly.

% NOTE: resulting srate needs to be dividible by 10 after downsampling

srate = 1000; % Sampling rate of interpolated sine wave

dwn = 40; % Downsampling factor

time = 390; % Total session time, in secs (for each condition)

% Design settings

ndyads = 14; % N of dyads

nsubs = 2; % N of subjects per dyad

nconds = 4; % N of conditions

nfeats = 10; % N of RQA features

ncycles = 10; % N of cycles

nsteps = 64; % N of metronome2 steps per cycle

%Condition labels

condlabels = {'Visual Coupling' , 'Visual Control' , 'Auditory Coupling' , 'Auditory Control'};

%% RP PARAMETERS (same parameters across all dyads)

% m (embedding dimension)

% tau (delay)

% ps (phase space size: we will set this at 10% of phase space)

path_home = ''; % set user's path (.../SI/Data processing_Matlab)

cd(path_home);

% Load data

load Dataset_S1.mat

% Pre-allocate parameters matrices for each dyad

[TAU, M, PS] = deal( NaN(ndyads, nsubs, nconds, ncycles) );

% Compute for each dyad

for dyadi = 1:ndyads

disp(['Computing paramenters: dyad #' num2str(dyadi)])

for subi = 1:nsubs %loop over partners within dyads

disp(['subject #' num2str(subi)])

for condi = 1:nconds % import for every condition

% Preprocessing of raw data (transfer discrete taps into a continuous sine wave,

% and downsample with factor defined in 'sets' structure)

disp(condlabels{condi})

% Interpolate long timeseries

eval(['[ onsets_tmp, ~] = interpolate_onsets(onsets_debounced(dyadi).sub' num2str(subi) '{condi});'])

% Down-sample sine wave

onsets_sine = downsample( onsets_tmp, dwn );

% Divide in cycles

onsets_sine = reshape ( onsets_sine , [] , ncycles );

% Define embedding parameters

for cycli = 1:ncycles % Compute for each cycle

disp(['Cycle #' num2str(cycli)])

% Exclude NaNs contained at extremes of cycle 1 and 10

[tau, m, ps] = RPparam(onsets_sine(~isnan(onsets_sine(:,cycli)),cycli), 1); %Set mFlag to 1 to output dimensionality m

% Store in dedicated matrices

TAU(subi, condi, dyadi, cycli) = tau;

M (subi, condi, dyadi, cycli) = m;

PS (subi, condi, dyadi, cycli) = ps;

end

end

end

end

% Calculate overall tau and ps

tau = round(mean(mean(mean(mean(TAU)))));

m = round(mean(mean(mean(mean(M)))));

ps = mean(mean(mean(mean(PS))));

% NOTE: median is not a linear operator: we cannot just

% neglect the order iterating the operation; see "median of medians

% problem". With the mean, there is no problem.

clearvars -except tau m ps TAU M PS

%% Timeseries pre-processing

path_home = ''; % set user's path (.../SI/Data processing_Matlab)

cd(path_home);

% Load raw data (de-bounced tapping onsets)

load Dataset_S1.mat % participants onsets

load Dataset_S2.mat % metronomes onsets

% Pre-allocate interpolated timeseries (to guarantee same vector length)

[sine_s1,sine_s2,saw_s1,saw_s2] = deal(nan( (srate/dwn)*time, ndyads, nconds));

% Interpolate metronome1

[~, temp_saw_m1] = interpolate_onsets(onsets_metro1); % interpolate sinewave

saw_m1 = downsample( temp_saw_m1, dwn ); %downsample ramp wave

% Repeat for metronome2

[~, temp_saw_m2] = interpolate_onsets(onsets_metro2); % interpolate sinewave

saw_m2 = downsample( temp_saw_m1, dwn ); %downsample ramp wave

%Process input data

for dyadi=1:ndyads

for condi=1:nconds

display(['Processing dyad = ' num2str(dyadi)]); display(['condition = ' num2str(condi)]);

% Interpolate subject1

[temp_sine_s1, temp_saw_s1] = interpolate_onsets(onsets_debounced(dyadi).sub1{condi}); % interpolate sinewave

sine_s1(:,dyadi,condi) = downsample( temp_sine_s1, dwn ); % downsample sinewave (too heavy for JRQA)

saw_s1(:,dyadi,condi) = downsample( temp_saw_s1, dwn );

% Repeat for subject2

[temp_sine_s2, temp_saw_s2] = interpolate_onsets(onsets_debounced(dyadi).sub2{condi}); % interpolate sinewave

sine_s2(:,dyadi,condi) = downsample( temp_sine_s2, dwn ); % downsample sinewave (too heavy for JRQA)

saw_s2(:,dyadi,condi) = downsample( temp_saw_s2, dwn );

end

end

% Replace the NaNs with a random position on the sinewave: jrqa() does not

% accept NaNs; this way, not joint recurrence can be computed where values

% are randomized.

randomizer1 = -rand(length(sine_s1(find(isnan(sine_s1)))),1) +rand(length(sine_s1(find(isnan(sine_s1)))),1);

randomizer2 = -rand(length(sine_s2(find(isnan(sine_s2)))),1) +rand(length(sine_s2(find(isnan(sine_s2)))),1);

sine_s1(find(isnan(sine_s1))) = randomizer1;

sine_s2(find(isnan(sine_s2))) = randomizer2;

% Compute relative phase

rf_expected = saw_m1-saw_m2; % Expected relative-phase (downsample)

rf_expected(rf_expected<0) = rf_expected(rf_expected<0)+2*pi; %"correct" negative values

rf_observed = saw_s1-saw_s2; % Observed relative phase

rf_observed(rf_observed<0) = rf_observed(rf_observed<0)+2*pi; %"correct" negative values

%% Cycle aggregation and recurrences

close all

% Initialize aggregated mesures

% Angular measures (mean module and angle)

[r , theta] = deal( zeros( length(sine_s1)/ncycles, ndyads, nconds ) );

% Recurrence measures

RP_aggr = cell(nconds,1); % Recurrence plot (2-D)

density = cell(nconds,1); % Density plot (1-D), looping over cols, summing all rows

r_score = zeros(nsteps,ndyads,nconds); % Response variable for LME model (split & average density plot)

% Embedding parameters - HARD-CODED as outputted by previous block (for srate=1000 and dwn=40)

m = 3; %number of dimentions

tau = 7; %number of time lags

ps = 0.3388; %phase-space size (10% of total phase space)

% Compute

for dyadi = 1:ndyads

disp(['Computing dyad #', num2str(dyadi) ' :'])

for condi = 1:nconds

disp(condlabels{condi})

% Divide relative phase in cycles

relph_single = reshape(rf_observed(:,dyadi,condi),[],ncycles);

sine_single1 = reshape(sine_s1(:,dyadi,condi),[],ncycles);

sine_single2 = reshape(sine_s2(:,dyadi,condi),[],ncycles);

% Compute vector length along second dimention (i.e., over all trials)

r(:,dyadi,condi) = circ_r(relph_single, [], [], 2);

% Compute mean angle

theta(:,dyadi,condi) = circ_mean(relph_single, [], 2); %add pi to re-scale to 2pi

% Compute Joint Recurrence Plot (JRP) for every cycle

for cycli = 1:ncycles

RP_temp = jrp(sine_single1(:,cycli), sine_single2(:,cycli), m, tau, ps);

% Overlap JRPs and sum

if cycli == 1

RP_aggr{condi} = RP_temp;

else

RP_aggr{condi} = RP_aggr{condi} + RP_temp; %Sum up RPs one at a time

end

end

% Compute 1-D density curve of recurrence points

for i = 1:length(RP_aggr{condi}) %loop over columns of the RP

density{condi}(i,1) = sum(RP_aggr{condi}(:,i)); %sum all rows

end

% Split density curve by metronome steps

steps = round(length(density{condi})/nsteps) * [1:nsteps];

% Average within windows

r_score(1,dyadi,condi) = mean(density{condi}(1:steps(1))); % from first point to first edge

for i = 2:length(steps)

r_score(i,dyadi,condi) = mean(density{condi}(steps(i-1):steps(i)));

end

end

end

%% Export RQA measures to R

%Initialize output table

outScore = [];

%Fill output table

for condi = 1:nconds

tempScore = [];

for dyadi = 1:ndyads

tempScore = [tempScore ; r_score(:,dyadi,condi)'];

end

outScore = [outScore ; tempScore];

end

%Export to Excel table

cd(path_home)

writematrix(outScore , 'rscore_temp.xls');

% Temporary file, includes response variable only;

% values are to be pasted in 'rscore_long.xlsx',

% which is ready to be imported in R for statistical analyses.

%% Visualization

% Pick the dyad to visualize

for dyadi = 5 %1:ndyads

for condi = 1:nconds

% Aggregated measures from relative phase

figure(200+dyadi); % Measures NOT included in the paper

subplot(211)

plot(r(:,dyadi,condi) , 'LineWidth' , 1.2)

hold on

title('Aggregated modules')

xticks([])

legend(condlabels)

subplot(212)

plot(theta(:,dyadi,condi) , 'LineWidth' , 1.2)

hold on

yline(0,'--');

title('Aggregated mean angles')

xticks([])

legend(condlabels)

% Recurrence plots and density scores

figure(300+dyadi)

%RPs

subplot(6,2,condi)

imagesc(rot90(RP_aggr{condi}))

colormap('parula')

%surf(double(rot90(RP_aggr{condi})) - rot90(ncycles*eye(size(RP_aggr{condi}))))

axis square

title(condlabels{condi})

xticks([]), yticks([])

%Recurrence score plot

subplot(6,2,condi+4)

plot(density{condi})

hold on

xline(round(length(density{condi})/2),'--');

axis square

title(condlabels{condi})

xticks([])

xlabel('Aggregated trial'), ylabel('Rp score')

ylim([0 max([density{1};density{2}])])

%Output for mixed-effects model

subplot(6,2,condi+8)

plot(r_score(:,dyadi,condi))

hold on

xline(round(nsteps/2),'--');

axis square

title(condlabels{condi})

xticks([])

xlabel('Aggregated trial'), ylabel('Rp score')

ylim([0 max([density{1};density{2}])])

end

end

hold off
