# Supplementary material for: Modality-specific attractor dynamics in dyadic entrainment
Source: Sci Rep. 2021 Sep 15;11:18355. doi: 10.1038/s41598-021-96054-8 (PMC8443558; doi:10.1038/s41598-021-96054-8)
Supplement: Supplementary file 7 — Supplementary Information 6. [file 41598_2021_96054_MOESM7_ESM.docx]

### This R script runs growth curve analysis (Mirman, 2017) on the recurrence score data from the ‘drifting metronomes’ paradigm. ###

# Import libraries

library(readxl)

library(ggplot2)

library(broom)

library(dplyr)

# Statistical package

library(lme4)

# Data arrangement

library(reshape2) # contains melt() function for long univariate format

library(ez)

library(psych)

### Normality tests ###

library(EnvStats)

### Colors in plot ###

library(RColorBrewer)

library(wesanderson)

### Tables for publication ###

library(sjPlot)

library(gridExtra)

# Import recurrence data (set user's path before file name)

recurrence <- read_excel('/Dataset_S3.xlsx')

# Convert variables to factors

recurrence$Modality <- factor(recurrence$Modality)

recurrence$Coupling <- factor(recurrence$Coupling)

# Wait to convert recurrence$Dyad: after first plotting in order to compute stats on numeric var

# Re-order factors, for interpretation

recurrence$Coupling <- relevel(recurrence$Coupling , 'Uncoupled')

contrasts(recurrence$Coupling) #control needs to be 0 -> intercept provides the baseline

recurrence$Modality <- relevel(recurrence$Modality , 'Auditory')

contrasts(recurrence$Modality) #set baseline for contrast across modalities

# Store number of steps

nsteps <- as.numeric(tail(names(recurrence), n=1)); #convert last variable name to numeric

# Convert to longitudinal data format: person, (other factors) , period

recurrence = melt(recurrence , id = c("Dyad","Coupling","Modality"))

# Rename serial position

recurrence <- recurrence %>% rename("Serial" = "variable")

recurrence <- recurrence %>% rename("Recurrence" = "value")

# Check long format

# summary(recurrence)

# Transform response variable

# (remember that if you fit on transformed data you have to re-convert the

# estimate coefficient at the end, for interpretation)

recurrence$Recurrence <- log(recurrence$Recurrence)

#Create higher-order orthogonal polynomial

npols <- 4 #number of polynomials

t <- poly(unique(1:nsteps),npols) #assign to 'time'; metronome clicks over cycle

#Create time variable in data frame

recurrence[,paste("ot",1:npols,sep="")] <-

t[recurrence$Serial, 1:npols]

# #Visualize polynomial

# ggplot(data = recurrence , aes(Serial , ot4)) + geom_point() +

# facet_grid() + #this is how you divide by subject

# #theme(axis.text.x = element_blank()) +

# theme(axis.text.y = element_blank()) +

# xlab("Expected relative phase") +

# ylab("Response variable") +

# scale_x_discrete(breaks=c("1","32","64"),

# labels=c("0", expression(pi), expression("2" ~ pi)))

### Model fitting ###

#Fit quadratic model

recurrence.quad <- lmer(Recurrence ~ (ot1+ot2)*Modality*Coupling +

(ot1+ot2 | Dyad) + #random effect of dyads

(ot1+ot2 | Dyad:Modality:Coupling) , #...and interactions with factors

control = lmerControl(optimizer = "bobyqa") ,

data = recurrence, REML=FALSE)

# final argument: if false, uses maximum likelihood estimation to fit the model

# (vs restricted maximum likelihood estimation)

#Fit quartic model

# recurrence.quart <- lmer(Recurrence ~ (ot1+ot2+ot3+ot4)*Modality*Coupling +

# (ot1+ot2+ot3+ot4 | Dyad) + #random effect of subjects

# (ot1+ot2+ot3+ot4 | Dyad:Modality:Coupling) ,

# control = lmerControl(optimizer = "bobyqa") ,

# data = recurrence, REML=FALSE)

#Get parameter estimates for all models

quad.coefs <- data.frame(coef(summary(recurrence.quad)))

#quart.coefs <- data.frame(coef(summary(recurrence.quart)))

#...and estimate p-values

quad.coefs$p <-

2*(1-pnorm(abs(quad.coefs$t.value)))

# quart.coefs$p <-

# 2*(1-pnorm(abs(quart.coefs$t.value)))

# Compute residuals

quad.res <- resid(recurrence.quad)

#quart.res <- resid(recurrence.quart)

# Inspect residuals

hist(quad.res)

qqnorm(quad.res, pch = 1, frame = FALSE)

qqline(quad.res, col = "steelblue", lwd = 2)

# Visualize grand-average per conditions

ggplot(data = recurrence , aes(x = Serial, y = Recurrence, color = Modality, shape = Coupling)) +

facet_grid(rows = vars(Modality) , cols = vars(Coupling)) +

geom_point() +

scale_color_brewer(palette="Set1") + #manually set color mapping

scale_shape_manual(values=c(16, 1))+ #manually set shape mapping

stat_summary(fun = mean , geom = "line", size = 2, color = 'black') + #mean timecourse

stat_summary(fun.data = mean_se, geom = "pointrange", color = 'black', alpha = 1) + #st error time course

xlab("Expected relative phase") +

ylab("Response variable") +

scale_x_discrete(breaks=c("1","32","64"),

labels=c("0", expression(pi), expression("2"~pi~" ")))

# Visualize fit per conditions

ggplot(data = recurrence , aes(x = Serial, y = fitted(recurrence.quad), color = Modality, shape = Coupling)) +

facet_grid(rows = vars(Modality) , cols = vars(Coupling)) +

geom_point() +

scale_color_brewer(palette="Set1") + #manually set color mapping

scale_shape_manual(values=c(16, 1))+ #manually set shape mapping

stat_summary(fun = mean , geom = "point", size = 2, color = 'black') + #mean timecourse

stat_summary(fun.data = mean_se, geom = "pointrange", color = 'black', alpha = 1) + #st error time course

xlab("Expected relative phase") +

ylab("Recurrence score (log scale)") +

scale_x_discrete(breaks=c("1","32","64"),

labels=c("0", expression(pi), expression("2"~pi~" ")))

# Visualize per dyad; x = ot1 because it's the linear increase in the model

paste("0",recurrence$Dyad[recurrence$Dyad<10], sep = "", collapse = NULL) # Re-label dyads, for proper order

ggplot(data = recurrence , aes(x = ot1, y = Recurrence , color = Modality , shape = Coupling)) +

geom_point() +

geom_line(aes(y = fitted(recurrence.quad))) +

scale_color_brewer(palette="Set1") + #manually set color mapping

scale_shape_manual(values=c(16, 1))+ #manually set shape mapping

facet_wrap(~Dyad) + #this is how you divide by subject

theme(axis.text.x = element_blank()) +

xlab("Expected relative phase") +

ylab("Recurrence score (log scale)")

#Inspect results

quad.coefs

#quart.coefs

# # Generate table

# table.model <- tab_model(recurrence.quad , show.se = TRUE ,show.ci = FALSE ,

# show.re.var = FALSE , show.obs = FALSE ,

# show.r2 = FALSE , show.icc = FALSE , show.ngroups = FALSE ,

# p.style = "numeric_star" ,

# pred.labels = c("(Intercept)","Time","Time^2","Modality","Coupling","Time:Modality","Time^2:Modality","Time:Coupling","Time^2:Coupling","Modality:Coupling","Time:Modality:Coupling","Time^2:Modality:Coupling"))

# # Save table

# ggsave(grid.table(table.model), filename = "~/Users/mattiaipem/Desktop/trytab.png")
