# Supplementary material for: Modality-specific attractor dynamics in dyadic entrainment
Source: Sci Rep. 2021 Sep 15;11:18355. doi: 10.1038/s41598-021-96054-8 (PMC8443558; doi:10.1038/s41598-021-96054-8)
Supplement: Supplementary file 8 — Supplementary Information 7. [file 41598_2021_96054_MOESM8_ESM.pdf]

Dyad ID ______ **Subject ID ______** **Condition ID ______** **Date:**

Please rate how much you agree with the following statements related to the task you just performed with your partner, on a scale from 1 (completely disagree) to 7 (completely agree).

During the last task:

1. I experienced a sense of drowsiness

   COMPLETELY DISAGREE — COMPLETELY AGREE

   1 2 3 4 5 6 7

2. I found it difficult to keep the tempo of my metronome

   COMPLETELY DISAGREE — COMPLETELY AGREE

   1 2 3 4 5 6 7

3. The task was more difficult than expected

   COMPLETELY DISAGREE — COMPLETELY AGREE

   1 2 3 4 5 6 7

4. The actions of my partner were affecting my actions

   COMPLETELY DISAGREE — COMPLETELY AGREE

   1 2 3 4 5 6 7

5. My actions were affecting the actions of my partner

   COMPLETELY DISAGREE — COMPLETELY AGREE

   1 2 3 4 5 6 7

6. I felt in control over my own actions

COMPLETELY DISAGREE — COMPLETELY AGREE

1 2 3 4 5 6 7

7. I felt like I was cooperating with my partner

COMPLETELY DISAGREE — COMPLETELY AGREE

1 2 3 4 5 6 7

8. I felt like I was competing with my partner

COMPLETELY DISAGREE — COMPLETELY AGREE

1 2 3 4 5 6 7

9. I experienced a sense of frustration

COMPLETELY DISAGREE — COMPLETELY AGREE

1 2 3 4 5 6 7

10. I felt like my actions were affected by what I was seeing

COMPLETELY DISAGREE — COMPLETELY AGREE

1 2 3 4 5 6 7

11. I felt like my actions were affected by what I was hearing

COMPLETELY DISAGREE — COMPLETELY AGREE

1 2 3 4 5 6 7

If you have any additional observation regarding your experience of the task, or any particular remarks about the experiment up to this point, you can write a short comment below.
